# Long-Term Exposure of MoS_2_ to Oxygen and Water Promoted Armchair-to-Zigzag-Directional Line Unzippings

**DOI:** 10.3390/nano12101706

**Published:** 2022-05-17

**Authors:** Youngho Song, Minsuk Park, Junmo Park, Hyun S. Ahn, Tae Kyu Kim, Sang-Yong Ju

**Affiliations:** Department of Chemistry, Yonsei University, Seoul 03722, Korea; yh05song@yonsei.ac.kr (Y.S.); min4218@yonsei.ac.kr (M.P.); parkjunmo2000@yonsei.ac.kr (J.P.); ahnhs@yonsei.ac.kr (H.S.A.); tkkim@yonsei.ac.kr (T.K.K.)

**Keywords:** molybdenum disulfide, line defect, unzipping, zigzag, oxygen

## Abstract

Understanding the long-term stability of MoS_2_ is important for various optoelectronic applications. Herein, we show that the long-term exposure to an oxygen atmosphere for up to a few months results in zigzag (zz)-directional line unzipping of the MoS_2_ basal plane. In contrast to exposure to dry or humid N_2_ atmospheres, dry O_2_ treatment promotes the initial formation of line defects, mainly along the armchair (ac) direction, and humid O_2_ treatment further promotes ac line unzipping near edges. Further incubation of MoS_2_ for a few months in an O_2_ atmosphere results in massive zz-directional line unzipping. The photoluminescence and the strain-doping plot based on two prominent bands in the Raman spectrum show that, in contrast to dry-N_2_-treated MoS_2_, the O_2_-treated MoS_2_ primarily exhibits hole doping, whereas humid-O_2_-treated MoS_2_ mainly exists in a neutral charge state with tension. This study provides a guideline for MoS_2_ preservation and a further method for generating controlled defects.

## 1. Introduction

Molybdenum disulfide (MoS_2_) is a representative of the transition metal dichalcogenide (TMDC) MX_2_ family which has sandwiched layer structures with the transition metal M (groups four, five, and six atoms) located between chalcogen atoms X (S, Se, and Te). Owing to the presence of a band gap associated with its few-atom-thick layers held together by van der Waals forces, MoS_2_ possesses interesting optical [1,2,3], spin–valley polarization [4,5], and catalytic properties [6,7,8]. In the context of its optical properties, neutral MoS_2_ displays two features known as A (~1.89 eV) and B (~2.08 eV) excitons [1,2,9], which are associated with direct transitions from the highest spin-split valence bands to the lowest conduction bands. Furthermore, the A exciton has subcomponents in the form of a charge-neutral exciton band A^o^ at 1.89 eV and a lower-lying charged exciton (trion) band A^−^ at 1.86 eV, whose relative intensities and positions are dependent on the doping [10,11,12] and strain (*ε*) [11,13,14,15,16] of MoS_2_. Applications of the optoelectronic properties of MoS_2_ require an understanding of its long-term stability.

MoS_2_ is amenable to both electron (*n*) or hole (*p*) doping [10,11] and it can possess strain (*ε*) [11,13,14,15,16]. The effects of environmentally abundant oxygen and water on the optoelectronic properties of MoS_2_ have been studied [17,18,19], but how these species affect the doping and *ε* properties of MoS_2_ under ambient conditions remains largely unknown, and this is an important issue. Treatments under harsh oxidative conditions such as with oxygen plasma [18], UV-ozone [20], and high-temperature annealing (>300 °C) [18,21,22] are known to induce oxidation of the basal plane of MoS_2_. Following these treatments, the basal plane of MoS_2_ exhibits reduced photoluminescence (PL), while edges and cracked regions display increased PL, suggesting that oxidation plays different roles in governing the properties of the basal plane and periphery. Moreover, O_2_ incubation of up to one year leads to random cracks and defects [19]. In contrast, physisorption of oxygen by MoS_2_ activated at 450 °C under vacuum results in a large basal-plane PL enhancement [17] caused by MoS_2_-to-O_2_ charge transfer. Because chemisorbed oxygen modulates the optical properties of MoS_2_ differently from physisorbed oxygen, it is important to gain an understanding of how chemical oxidation controls the doping and *ε*, which in turn govern the band-gap structure of MoS_2_ [12].

Theoretical study [23] suggests that the basal plane of MoS_2_ exhibits a large kinetic barrier (i.e., 1.6 eV) for O_2_ chemisorption, whereas vacancies (i.e., sulfur vacancies) at the surface of MoS_2_ reduce the barrier to 0.8 eV. The experimentally determined activation energy for bulk MoS_2_ oxidation (0.54 eV and 0.79 eV) [22,24] is somewhat lower than the theoretically predicted value. However, recent scanning tunneling microscope measurements [25] show that point-like oxygen-substitution reactions producing oxygenated MoS_2_ occur spontaneously, even under ambient conditions. Along with the fact that Mo-terminated edges of MoS_2_ readily react with O_2_ [26], these findings indicate that oxygenated MoS_2_ possesses various point defects which are randomly distributed over the plane.

The investigation described below was designed to address this issue. For this purpose, chemical vapor deposition (CVD)-grown MoS_2_ crystals were exposed to four different conditions for two weeks: N_2_, N_2_ with 75% relative humidity (N_2_-75RH), O_2_, and O_2_ with 75% relative humidity (O_2_-75RH). Using various methods, including Raman spectroscopy, photoluminescence (PL) spectroscopy, and atomic force microscopy (AFM), we observed that the basal plane of MoS_2_ possessing tensile strain (*ε*_T_) associated with its preparation using CVD, undergoes zigzag (zz)-directional unzipping upon long-term exposure to an oxygen atmosphere. Specifically, during the initial phases of exposure to O_2_ and O_2_-75RH, MoS_2_ crystals display initial macroscopic armchair (ac)-directional micrometer-scale defects near triangular edges. Following increased exposure times of up to a few months, the initial ac-directional defects near the periphery change into zz-directional unzipping in the basal plane. This unique unzipping behavior is associated with the susceptibility of S defects in the basal plane to tension caused by oxidation. Moreover, Raman and PL spectroscopic studies show that changes occurring in the optoelectronic properties of MoS_2_ upon chemical oxidation are a consequence of changes in *ε*_T_ and doping.

## 2. Materials and Methods

### 2.1. Materials and Instrumentation

MoO_3_ (product no. 267856, ACS reagents, purity ≥ 99.5%) and sulfur (product no. 13803, purity ≥ 99.5%) were purchased from Sigma-Aldrich (St. Louis, MO, USA). Sulfur was further recrystallized using vacuum sublimation at ca. 10^−3^ torr, as described previously [27]. Sodium cholate (SC), with a purity of over 98%, was purchased from TCI (Tokyo, Japan) and used as a surfactant and an adhesion promoter to a silicon substrate. Deionized water with a resistivity greater than 18 MΩ was used. All gases, including N_2_, Ar, and O_2_ with purities of over 99.99% were obtained from Donga Gas (Jinju, Korea). The 285-nm thick SiO_2_/Si substrates (lot no. 7400397-601, Shin-Etsu, Tokyo, Japan) were spin-coated with MoO_3_ and converted to MoS_2_. The as-received wafer was cut into pieces of size 1.0 × 1.0 cm^2^ and further rinsed with methanol, acetone, and isopropanol while undergoing bath sonication, then subjected to drying with a N_2_ stream. Optical microscope (OM) images were obtained using an upright fluorescence microscope (BX-51, Olympus, Tokyo, Japan) with a CMOS camera (3.6 μm/pixel, 1280 × 1024 pixels, part no. DCC1645C, Thorlabs, Newton, NJ, USA) and a light-emitting diode light source (cold white color, part no. MCWHL2, Thorlabs) with an optional dichroic cube set (MWG, Olympus), which could image the 660 nm PL of MoS_2_. SEM images were acquired using field-emission SEMs (SU8000, Hitachi or 7610f-plus, JEOL Ltd., Tokyo, Japan) operating at an acceleration voltage of 5 kV.

### 2.2. Precursor Preparation

MoS_2_ growth involved the use of SC as adhesion promoter and surfactant for MoO_3_ powder, according to the procedure described in the literature [28]. Aqueous MoO_3_ dispersion was prepared by sonication using SC as a surfactant. Briefly, 20 mM MoO_3_ was added to 1 wt. % SC in 100 mL of water. The suspension was subjected to bath sonication for 1 h (70 W, Branson1519, Brookfield, CT, USA) followed by tip sonication for 2 h (300 W amplitude, probe tip diameter: 13 mm, VCX 750, Sonics and Materials, Newtown, CT, USA). The dispersion was centrifuged using a table-top centrifuge (Wisespin CF-10, Daihan Scientific Co, Ltd., Wonju-si, Korea) to collect an 80% supernatant. 100 μL of the MoO_3_ dispersion was spin-coated at 3000 rpm for 80 s by drop-casting on precleaned SiO_2_ on a Si substrate. Sulfur was prepared by vacuum sublimation. Briefly, 1 g of sulfur was placed at the bottom of a sublimation kit with a cold finger. The sublimation temperature was set to 200 °C under vacuum (10^−3^ torr).

### 2.3. Growth of MoS_2_ Crystal

MoS_2_ was prepared by using a hot-wall CVD apparatus [29] operating at atmospheric pressure, with the aforementioned spin-coated MoO_3_ and the sublimed sulfur powder as precursors, modified from the previous study [27]. Prior to the MoS_2_ growth, a quartz tube in a tube furnace was pre-annealed for 30 min at 1000 °C with a 20 standard cubic centimeter per min (sccm) Ar flow to remove any physisorbed water, in a CVD chamber. After cooling, the MoO_3_ spin-coated substrate was loaded into the hot zone of a tube furnace with the SiO_2_ side face up, and an alumina boat containing 30 mg of sulfur was placed in an upstream position. Crystal growth was conducted at 750 °C for 20 min, at which time the alumina boat containing the sulfur reached ~220 °C. The substrate was then cooled to room temperature.

### 2.4. Environmental Control of MoS_2_

Four different environments (i.e., N_2_, N_2_-75RH, O_2_, and O_2_-75RH) were prepared using a Schlenk line technique and a septum-capped vial, with precautions as follows. For the N_2_-75RH or O_2_-75RH environments, a vial containing saturated brine solution maintaining 75RH [30] was bubbled with the N_2_ (or O_2_) for 90 min using a syringe to remove any O_2_ dissolved in the solution. For the N_2_ or O_2_ environments, a vial containing a moisture-absorbing silica gel was initially flame-dried thoroughly with a hand-held torch while a vacuum (10^−3^ torr) was pulled, and the sample was subsequently treated with a cycle of N_2_ (or O_2_) purging/vacuum, at least four times. Each sample was incubated for two weeks prior to any measurements. A further one month of incubation was conducted for observation of long-term changes.

### 2.5. Raman and PL Measurements

The samples were loaded into an environmental chamber (TS1000V-17/3 with T96-S, Linkam Scientific Instruments Ltd., Redhill, UK) which allowed observation of the sample through a coverslip while measurements were made. Raman and PL spectra were obtained using a micro-Raman spectroscopy setup with a backscattering geometry, as described in the literature [27,29,31]. Briefly, a spectrometer (Triax 320 with 1800 gr/mm) and coverslip-tolerant 40× objective (UPlanSApo, N.A.: 0.95, Olympus) were utilized to obtain the Raman and PL spectra. Calibration for Raman spectroscopy was conducted with multiple Hg/Ar lamp peaks using a light source (HG-1, Ocean Optics, Oxford, UK), according to the procedure described in the literature [29]. The Si peak at 520.89 cm^−1^ was used as an internal reference and an intensity-normalizing peak. Laser power was maintained below 0.06 mW to minimize any light-induced damage. The obtained Raman and PL spectra were further deconvoluted with Lorentzian fitting. Especially in the PL spectra, A°, A^−^, and B excitons were fitted to an unrestricted position and area.

### 2.6. Construction of Coordination System for ε–n Plot

The Raman origin ((E^1^_2g_, A_1g_) = (385.3 cm^−1^, 404.8 cm^−1^)) for the *ε* and *n* plot was taken from the values derived from suspended MoS_2_ in the literature [11]. Biaxial *ε* vs. *n* coordinates were introduced by modification of the *ε*–*n* diagram described previously [32,33,34], as in the case of graphene [35]. Since CVD-grown MoS_2_ exhibits biaxial *ε*_T_ [36], biaxial *ε*_T_ and Raman responses were utilized [11]. The changes in in-plane vibration E^1^_2g_ (Δ*ω*_E_) and out-of-plane vibration A_1g_ (Δω_A_) and their variations were Δ*ω*_E_ = −5.2 cm^−1^/% and Δω_A_ = −1.7 cm^−1^/%. Using the formula *γ* = [*ω* − *ω*_0_]/[2*εω*_0_], we determined the Grüneisen parameters *γ* for the Raman modes to be *γ*_E^1^_2g__ = 0.68 and *γ*_A_1g__ = 0.21, with the slope per *ε* or Δ*ω*_A_/Δ*ω*_E_ = 0.35 [11]. In the case of *n*, vibrational changes in E^1^_2g_ and A_1g_ and their extents were Δ*ω*_E_ = 0.21 cm^−1^ and Δ*ω*_A_ = 1.97 cm^−1^ per 1 × 10^12^ cm^−2^, with the slope of *n* with Δ*ω*_A_/Δ*ω*_E_ = 9.4 [35].

### 2.7. AFM Measurements

AFM height and phase images were obtained by using a tapping mode with an NX-10 AFM (Park Systems, Suwon, Korea). Al-coated silicon cantilevers (force constant: 37 N/m, resonance frequency: 300 kHz, ACTA-20, App Nano, Mountain View, CA, USA) were used. Typically, 512 × 512 pixels for a 40 μm length were routinely acquired at a speed of 0.2 Hz. The XEI program (Park Systems, Korea) was used to flatten topographies along the fast axis of scan using a polynomial, by excluding speckles of size 5 nm.

## 3. Results

CVD-grown triangular MoS_2_ crystals were single crystals terminated with zz edges [37] and were used to probe the effects of oxidation reactions on the morphological and optoelectronic properties. The MoS_2_ crystals were grown by CVD, using the procedure developed in our previous investigation [27], starting with MoO_3_ and sublimed sulfur (see Materials and Methods section). First, a well-dispersed aqueous MoO_3_ dispersion containing 1 wt. % sodium cholate (SC) as a surfactant and adhesion promoter [28] was spin-coated onto a 285 nm thick SiO_2_/Si substrate. After annealing at 1000 °C to eliminate adsorbates, a quartz tube was loaded with the MoO_3_-coated substrate and a boat containing freshly sublimed sulfur. A 20 sccm Ar flow was used as a carrier gas and the temperature of the hot zone was raised to 750 °C for 20 min, to promote MoO_3_ reduction with sulfur. The growth of the MoS_2_ crystals was terminated by cooling the tube to room temperature for 40 min while maintaining the Ar flow.

**Figure 1:**
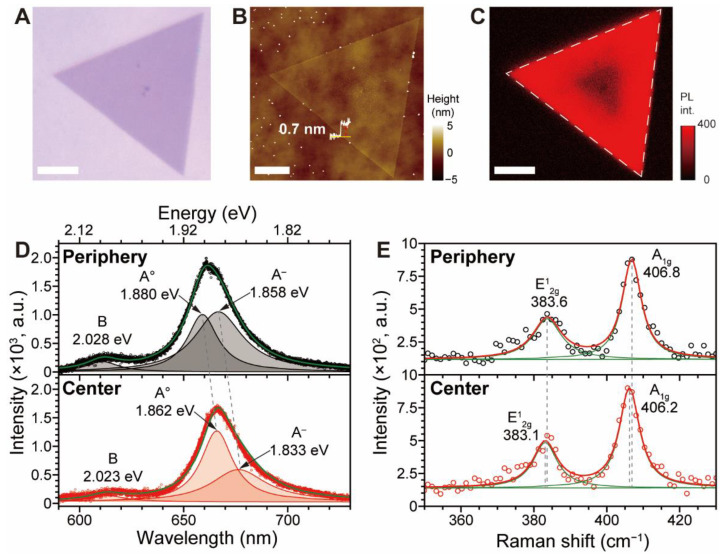
The characterizations of the as-grown MoS_2_ grains by various methods: (**A**) OM image; (**B**) the corresponding AFM height topography; (**C**) PL image with emission filtered using a 660 nm bandpass filter. Scale bars: 10 μm. (**D**) PL spectra obtained from central and peripheral parts of MoS_2_ and its Lorentzian deconvolutions to indicate A°, A^−^, and B, respectively. Dashed lines are drawn for comparison of the position. (**E**) The corresponding Raman spectra (circles) and its Lorentzian deconvolutions (green).

The Initial characterization of the as-grown MoS_2_ as a control was conducted using various methods, including optical microscopy (OM), atomic force microscopy (AFM), photoluminescence (PL) imaging/spectroscopy, and Raman spectroscopy (see Figure 1A–E). Inspection of the representative CVD-grown MoS_2_ via the OM image (Figure 1A) shows the MoS_2_ crystal as a ca. 46 μm long equilateral triangle with uniform contrast. The corresponding AFM height image (Figure 1B) shows that the crystal has a clean surface and a 0.70 nm edge height, indicating a monolayer of MoS_2_ [37]. Notably, the PL image (Figure 1C) shows a gradient of PL brightening from the center to the peripheral regions. Other researchers [16,36] have found that such gradual PL intensity (*I*_PL_) and peak position (*λ*) changes, as well as shifts in the Raman bands in CVD-grown MoS_2_, occur when proceeding from the center to the peripheral regions owing to differences in tensile strain (*ε*_T_) caused by thermal expansion coefficient differences between the Si substrate and the MoS_2_. Since biaxial *ε*_T_ shifts the excitonic A band by −99 meV/% [11], the observed *λ* of 675 nm in the PL spectrum at the center (Figure 1D) suggests the presence of substantial *ε*_T_ (i.e., 0.4%) compared to that at peripheral regions where the *λ* is ca. 660 nm. The positions of the two characteristic Raman bands of the in-plane vibration E^1^_2g_ and out-of-plane vibration A_1g_ are known to be sensitive to *ε*_T_, and E^1^_2g_ undergoes larger downfield shifts compared to A_1g_ with increasing *ε*_T_ [11,13,14,15,16,38]. The Raman spectrum of the central MoS_2_ region (Figure 1E) contains E^1^_2g_ at 383.1 cm^−1^ and A_1g_ at 406.2 cm^−1^ [39], whereas the spectra at the peripheries contain upshifted 383.6 cm^−1^ and 406.8 cm^−1^ bands, which is in good agreement with reported spectra of MoS_2_ under *ε*_T_. The results suggest that *ε*_T_ is a major contributor to the anisotropy present in the PL and Raman spectra of the as-prepared MoS_2_ crystal. Immediately after characterization, the MoS_2_ crystals were incubated under the four different atmospheres N_2_, N_2_-75RH, O_2_, and O_2_-75RH for two weeks (see Figure S1A in the Supplementary Materials (SM) for schematics of the environmental incubation processes).

**Figure 2:**
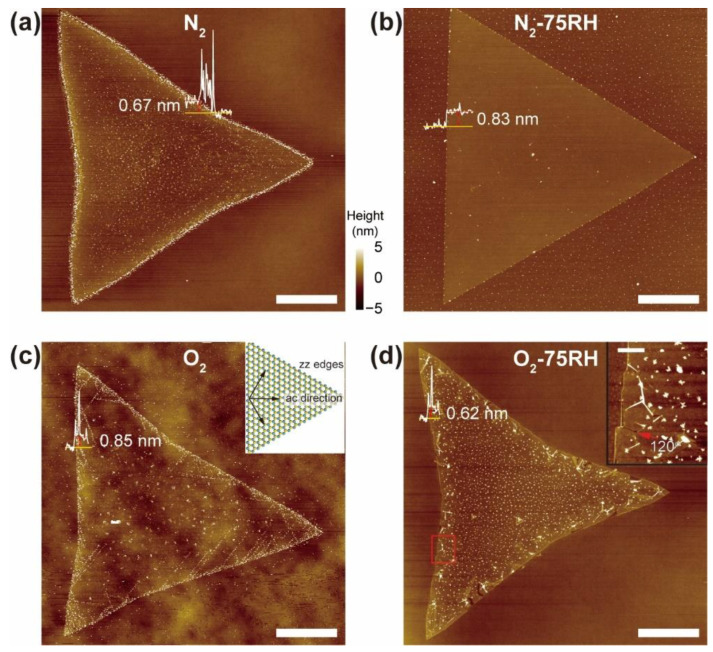
AFM height images of MoS_2_ crystals treated with: (**a**) N_2_; (**b**) N_2_-75RH; (**c**) O_2_; (**d**) O_2_-75RH. White traces are height profiles of MoS_2_. Inset of (**c**): crystallographic orientation of line defects of O_2_-treated MoS_2_, with Mo and S color-coded in green and yellow, respectively. Inset of (**d**): AFM height image of directional unzipping from MoS_2_ edges in red box in (**d**). Scale bar: 10 μm for (**a**–**d**); 1 μm for the inset of (**d**).

The effects of various environments on the morphology of MoS_2_ were first examined using AFM (Figure 2a–d). All crystals under the four conditions have triangular shapes and heights (white traces) varying from 0.6 to 0.85 nm, indicating monolayer MoS_2_. While the N_2_ and N_2_-75RH samples (Figure 2a,b) have topographies similar to the as-grown material, surprisingly, the MoS_2_ subjected to an O_2_ atmosphere (Figure 2c) displays few micrometer-long directional line defects originating from the edges. These defects are more visible in the phase image (Figure S2C,D), compared to those from N_2_-treated samples. Notably, the angles of the line defects against zz edges were ca. 30, 90, and 150° (see inset of Figure 2c), suggesting ac-directional line defects. This result contrasts with the report in [19] that aging of CVD-grown MoS_2_ and WS_2_ under ambient environmental conditions leads to random cracks and defects. This result suggests that the conditions for CVD growth affect the oxidative defects. Furthermore, the O_2_-75RH-treated sample (Figure 2d) shows that unzipping occurs from the edges in the ac directions. The inset in Figure 2d shows that unzipping, instead of occurring at ac line defects, occurs at the edges, and that crack directions are at 120° from each other (see red arrows for the intersection). This result suggests that dry and humid O_2_ oxidations promote stepwise transformations in the MoS_2_ crystal, from line defects to eventual unzipping.

**Figure 3:**
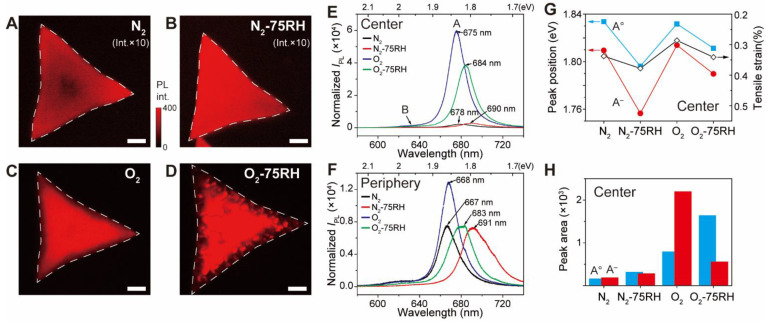
PL images, spectra, and analysis at central and peripheral positions of MoS_2_ crystals after incubation for two weeks under each condition. PL images from: (**A**) N_2_; (**B**) N_2_-75RH; (**C**) O_2_; (**D**) O_2_-75RH samples. Scale bar: 10 μm. PL intensities of (**A**,**B**) were multiplied for visual comparison. Normalized PL spectra from (**E**) central, and (**F**) peripheral regions with respect to the 520.89 cm^−1^ Si peak. (**G**) PL peak position and (**H**) PL peak-area changes of A^o^ and A^−^ derived by each treatment from central regions.

Because unzipping typically begins at the mechanically weakest points, the line defects and the subsequent unzipping are likely to be correlated with the *ε*_T_ of the MoS_2_, which in turn is associated with the optical properties of the MoS_2_ crystal. Figure 3A–D show the corresponding PL images of the samples. The images show that the *I*_PL_ for the N_2_-treated crystal gradually increases from the center to the periphery, suggesting that the *ε*_T_ behavior is similar to that of as-prepared MoS_2_. Interestingly, the N_2_-75RH sample (Figure 3B) has a uniform *I*_PL_ at both the center and periphery. A humid environment is known to form entrapped water between graphene and the substrate [40,41,42] or MoS_2_ and the substrate [43]. To confirm that such entrapped water is related to the uniform *I*_PL_, AFM height measurements of the basal plane from the N_2_-75RH sample (Figure S3A) were made. Water was entrapped evenly over the sample, and water entrapped regions is 0.5 nm thick and a few micrometers wide (Figure S3B) were entrapped between the MoS_2_ and the substrate. This was a phenomenon not seen in the N_2_-treated sample. These results are in agreement with the previous study, which demonstrated that a monolayer of water adhered under the MoS_2_ surface to a thickness of ~0.5 nm [43], as seen in other two-dimensional materials such as graphene [40,41,42]. This result suggests that the increased height induced by water and the subsequent strain might increase *ε*_T_ and lead to the observed uniform *I*_PL_ over the MoS_2_ crystal.

The PL image from the O_2_-treated sample (Figure 3C) shows that *I*_PL_ gradually increased from the periphery to the center, a trend that is opposite to that occurring in the N_2_-treated sample. The low *I*_PL_ near the periphery overlaps with the AFM-observed line defects present in the O_2_-treated MoS_2_. These results suggest that, unlike for samples treated using harsh oxidations [18,20,22], physisorbed O_2_ at the center of the crystal actually results in an enhancement of *I*_PL_, which is in accordance with the previous report [17]. In contrast, chemically oxygenated species evidenced by line defects form near the periphery and result in the reduced *I*_PL_. Similar to the O_2_ sample, the crystal incubated under the O_2_-75RH condition (Figure 3D) displays an *I*_PL_ that is brighter at the center and dimmer and more irregular at the periphery. Such spatial inhomogeneity originates from unzipping and folding of the MoS_2_, as evidenced by comparing the PL and AFM images.

Qualitative information was gained about doping and *ε*_T_ by analyzing the PL spectra from the central (Figure 3E) and peripheral (Figure 3F) regions of the incubated MoS_2_ crystals. Inspection of the spectra of the basal planes (Figure 3E) treated using humidified atmospheres (i.e., N_2_-75RH and O_2_-75RH) show that the *λ* values of the A bands display a bathochromic shift from 24 to 32 meV compared with those treated with N_2_ and O_2_ only. Moreover, the basal plane of the O_2_-treated sample exhibits an *I*_PL_ about 11 times larger than that of the crystal exposed to N_2_. The spectra from the peripheries (Figure 3F) show that while the peripheral *I*_PL_s of the O_2_ and O_2_-75RH samples were lower than those associated with the basal plane, the peripheral *I*_PL_ of the N_2_ sample was increased and that of N_2_-75RH was unchanged. These results suggest that the center and periphery of the MoS_2_ crystal experience different degrees of doping and *ε*_T_.

The *λ* and *I*_PL_ of A^o^ and A^−^ peaks in the PL spectra of MoS_2_ are dependent upon *ε* and *n*, and this serves as a foundation for deciphering the roles that O_2_ and H_2_O play in forming peripheral defects and unzipping [12,36]. To elucidate these values, the PL spectra at the central positions of the MoS_2_ crystals were deconvoluted using Lorentzians (shaded area), as shown in Figure S4. As evidenced by the dashed lines, the *λ* values and peak areas of A^o^ and A^−^ underwent a systematic change for crystals treated using each condition. For example, among the four samples, the dry N_2_- and O_2_-treated samples exhibited the most blue-shifted *λ* values for the A^o^ and A^−^ peaks, suggesting that they are associated with a smaller *ε*_T_. In addition, the *λ* values of the A^o^ and A^−^ (Figure 3G) bands of samples treated using humid atmospheres exhibited proportional red shifts. A comparison of the relative areas of the A^o^ and A^−^ bands (Figure 3F), which provides information about the charge state of MoS_2_ [10], shows that peak areas are much greater in the spectra of O_2_ and O_2_-75RH samples. Moreover, the comparison shows that water induces an increase in the population of the charge-neutral A^o^ state, and O_2_ causes an increase in the area of the charged A^−^ state. This result is opposite to that of a previous study suggesting that water has a *p*-doping effect [43], and suggests that water promotes neutral exciton formation while physisorbed O_2_ facilitates trion formation. Similar analysis of the PL spectra of peripheral regions (Figure S5A–C) shows that analogous but lesser shifts in *λ* occur (Figure S5B) and that the relative areas of A^o^ and A^−^ bands are relatively smaller compared to those in the spectra of basal planes. These observations in peripheral regions are in line with doping created by line defects and unzipping [19].

**Figure 4:**
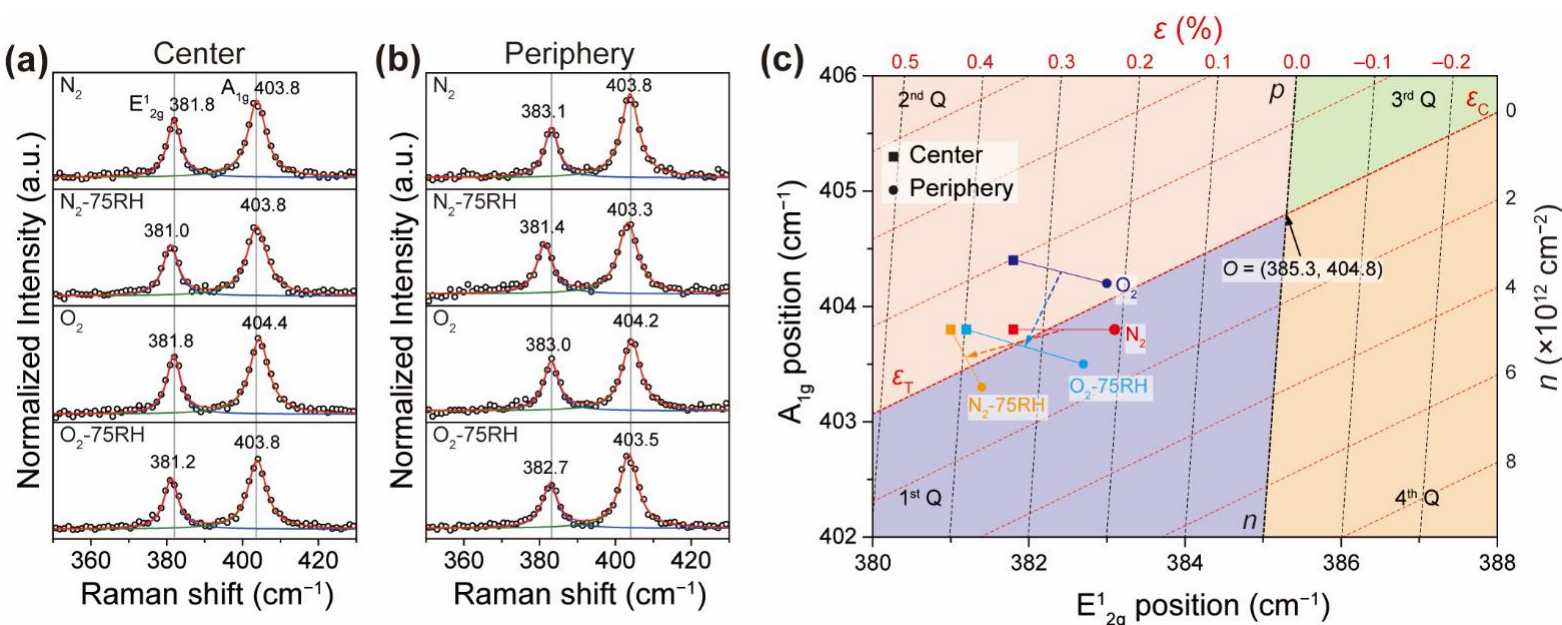
Raman spectra of (**a**) central and (**b**) peripheral regions from the incubated MoS_2_ crystals. Excitation wavelength: 532 nm. Raman spectra were deconvoluted by Lorentzian shape analysis and normalized according to the Si peak at 520.84 cm^–1^ nm. (**c**) Plot of *ε* (black dashed line) and *n* (red dashed line) with origin *O* = (E^1^_2g_, A_1g_) = (385.3, 404.8) extracted from Raman spectra of suspended MoS_2_ sample. Here, *ε*_T_ and *ε*_C_ stand for tensile and compressive strains, respectively, and *n* and *p* denote *n* and *p* doping, respectively. Solid lines between Raman points are drawn for grouping the same samples.

Raman spectroscopy is a powerful tool for gaining an understanding of the quantitative *ε* and *n*- or *p*-doping density of MoS_2_, because these parameters are closely related to chemical bond strengthening or weakening, which alters the vibrational behavior. A previous study [35] showed that *ε* and *n* contributions in graphene are quantitatively associated with two Raman bands (i.e., G and 2D modes). A similar concept has been utilized to evaluate *ε* and *n* for monolayer MoS_2_ [32,33,34]. To apply this treatment, we chose the frequencies from the spectra of suspended MoS_2_ as an origin for the unperturbed, pristine state [44,45]. Utilizing the published values for *ε* and *n* of monolayer MoS_2_ [11,46], an *ε*–*n* coordinate system in units of % and 1 × 10^12^ cm^−2^ (Figure 4c) was devised in a coordinate framework comprising two prominent Raman bands (E^1^_2g_, A_1g_). Specifically, Figure 4c is a plot of *ε* (black dashed line) and *n* (red dashed line), with an origin *O* = (E^1^_2g_, A_1g_) = (385.3, 404.8), corresponding to pristine MoS_2_. In addition, *ε*_C_ values are compressive strains, and *n* and *p* denote *n* and *p* doping, respectively. Representative crystal spectra acquired from the center and periphery are shown in Figure 4a,b. It is noteworthy that Raman bands near 275 cm^−1^ corresponding to MoO_3_ are not present [21]. As shown in Figure 4c, the central (peripheral) position of the N_2_ sample displays two bands at 381.8 (383.1) and 403.8 (403.8) cm^−1^. In terms of strain doping, the central part has *ε*–*n* coordinates of (0.34, −0.4), and the peripheral part has coordinates of (0.2, 0.7). This result suggests that while *ε*_T_ decreases from the center to the periphery, in accordance with previous findings [27,36], a slight *p*- to *n*-doping transition occurs simultaneously. The doping of the N_2_-treated sample is likely to be related to the interaction of the basal MoS_2_ with the SiO_2_/Si substrate, which acts as a *p* dopant [29,35]. Compared to the N_2_-treated sample, the N_2_-75RH sample (orange) exhibited much higher *ε*_T_ values (from 0.37 to 0.42%) at both the central and peripheral positions and experienced only a negligible doping density change. This result further supports the idea of the presence of water-induced tensile strain. This result underscores the advantage of utilizing an *ε*–*n* plot for analyzing the spectroscopic data, because otherwise the *p*-doping and *ε*_T_ results are both included in a similar downshift in vibrational frequencies [11].

Inspection of the *ε*–*n* plot shows that the two prominent Raman bands (E^1^_2g_ and A_1g_) associated with the central and peripheral regions of the O_2_- and N_2_-treated MoS_2_ crystals exhibited nearly similar movement along the *p*-doping axis. This observation suggests that O_2_ treatment results in *p*-doping, which is in agreement with the occurrence of charge transfer from MoS_2_ to physisorbed O_2_ [17]. Finally, the O_2_-75RH treated sample has *ε*–*n* coordinates of (0.4, −1) and (0.25, 1) for the central and peripheral positions, respectively. The different doping densities with different signs observed for the central and peripheral regions are closely connected to the existence of peripheral unzipping and folding, which decrease *ε*_T_. The increase in *ε*_T_ and the charge neutralization taking place in changing from O_2_ to O_2_-75RH, in conjunction with the PL results, indicate that treatments with O_2_ and H_2_O lead predominantly to *p*-doping and *ε*_T_, respectively.

**Figure 5:**
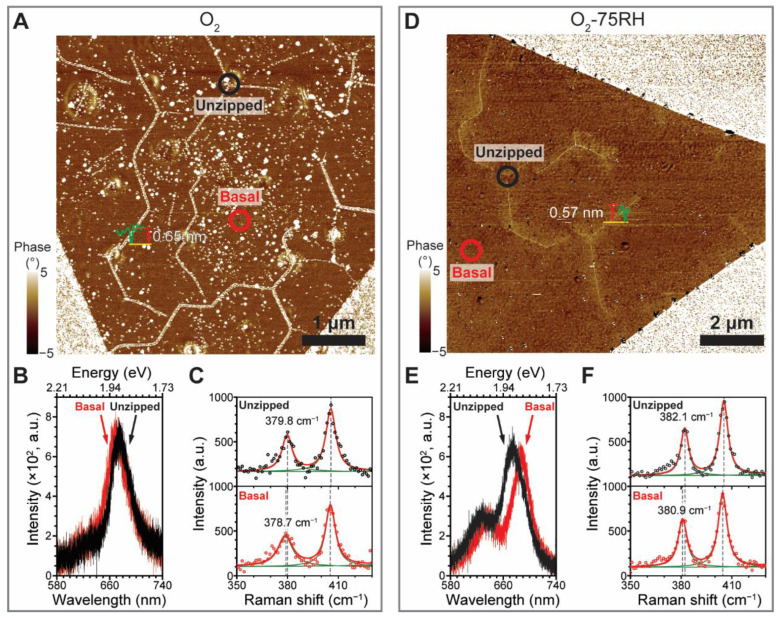
The zz-directional line unzipping of MoS_2_ induced by a three-month exposure to O_2_ and O_2_-75RH environments. (**A**) AFM phase image of MoS_2_ with O_2_. (**B**) Corresponding normalized PL spectra and (**C**) Raman spectra obtained from marked positions in (**A**). Line profile was obtained from the corresponding height image. Raman bands were deconvoluted by Lorentzian shape analysis. (**D**) AFM phase image of MoS_2_ after three-month incubations with O_2_-75RH. (**E**) Corresponding PL spectra and (**F**) Raman spectra obtained from marked positions in (**D**).

The results suggest that regardless of the environmental conditions used, MoS_2_ samples possess considerable *ε*_T_, albeit with different doping densities. However, only O_2_-treated crystals experienced line defects and unzipping. This observation prompted us to perform an experiment in which MoS_2_ crystals were exposed to O_2_ and O_2_-75RH environments for three months. Figure 5A,D show the respective AFM phase images, facilitating the visualization of unzipping. Remarkably, both samples show zz-directional line unzipping with respect to zz-terminated edges. The O_2_-treated sample had a wider unzipping width compared to the O_2_-75RH-treated MoS_2_. Unzippings occurred at 120° with respect to each other. Although unzipping near the periphery is ac-directional with respect to the edge, it changes to the zz direction in the basal plane. This finding stands in stark contrast to the etched triangular pit of exfoliated MoS_2_ prepared by high-temperature annealing (i.e., 300 °C) [21,47] and the random cracks incubated at room temperature [19]. Inspection of the normalized PL spectra of the O_2_-incubated crystal (Figure 5B) shows that both unzipping and basal positions occur at ~670 nm, which corresponds to the near-absence of tension. Similarly, both Raman spectra (Figure 5C) show similar interpeak separations (i.e., ~25 cm^−1^). A similar unzipping behavior but associated with a larger difference in *ε*_T_ was observed for the O_2_-75RH-treated sample. In this case, the PL peak position (Figure 5E) of the unzipped region (black circle in Figure 5D) displayed a large blue shift (20 nm) compared to that from the basal plane, showing that the *ε*_T_ was relieved. Raman spectra analysis (Figure 5F) further supported the fact that the basal plane has a larger *ε*_T_ (larger interpeak separation) compared to that for the O_2_-treated crystal.

These defects are different from the defects that can exist in the as-grown state. Figure S6A,B show PL images of the as-prepared MoS_2_ sample and the same sample after incubation. Bright PL originates from the crack or unzipping regions, showing an increase in line defects. However, the existing cracks in the as-prepared sample appear to be random, in contrast to the precise zz-directional unzipping after O_2_-75RH treatment. The AFM phase image (Figure S6C) clearly shows that the O_2_-75RH-treated sample had precise turns and angles of unzipping with respect to the edges. In addition, proceeding from the edges to the center slowly changed the unzipping direction from ac to zz lines. Figure S7 shows the AFM phase images from O_2_- (Figure S7A–C) and O_2_-75RH-treated (Figure S7D–F) samples. Irrespective of the presence of H_2_O, nearly all the ac unzipping at the edges changed into the zz direction in the basal plane within a few micrometers. Interestingly, the width of the ac unzipping is much less than that of the zz unzipping, presumably owing to the *ε*_T_ difference in the central and peripheral regions. Along with the PL and Raman studies, this finding strongly supports the fact that directional unzipping is dependent on *ε*.

**Figure 6:**
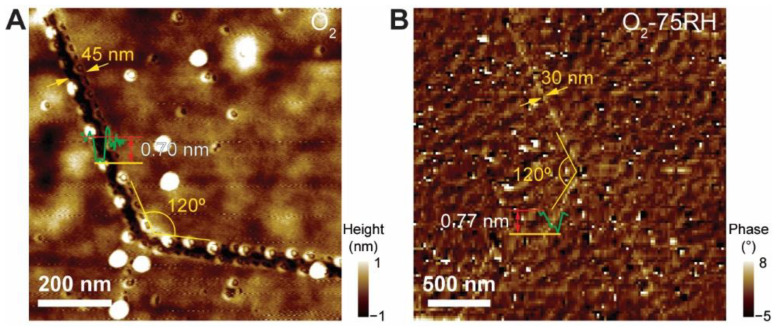
(**A**) AFM height image of line unzipping from O_2_-treated MoS_2_ sample. (**B**) AFM phase image of line unzipping from O_2_-75RH-treated MoS_2_ sample. It is noteworthy that the phase image was selected to display cracks. Line profile was obtained from the corresponding height image.

The AFM image (Figure 6A) reveals details of the zz-directional line unzipping and origin. The O_2_-treated sample shows 45 nm wide unzipping. The width is persistent along the unzipped segments. In addition, the 120° turns are very sharp. Since the observed typical *ε*_T_ is in the range of 0.2–0.4%, the width of the MoS_2_ grain extends by 20–40 nm, which accounts for the few unzippings with a 45 nm width. Similarly, the AFM phase image of the O_2_-75RH sample (Figure 6B), which has similar *ε*_T_, displays somewhat similar line width (i.e., 30 nm). As evidenced by Figure S7, typical unzipping occurs in two or three lines along the 10 μm wide MoS_2_ crystals, in good agreement with the observed line unzipping. Moreover, MoS_2_ with a few layers also has similar unzipping. Figure S7A–C shows the AFM and corresponding OM images of MoS_2_ with a few layers. The few-layered regions have less line unzipping compared to single-layered MoS_2_. In addition, line unzipping is much more random, as indicated by the yellow arrows. We speculate that this effect of fewer and more random unzippings originates from the lesser *ε*_T_ exerted on few-layered MoS_2_.

The remaining question to be addressed is that of how O_2_ treatment causes MoS_2_ to unzip along the zz directions. Raman spectroscopic analysis did not show the presence of detectable signals associated with MoO_3_. In addition, the energy dispersive spectrum (EDS) using scanning electron microscopy (SEM) was also used to attempt to probe the nature of line defects, and this only showed strong Si and O signals from the substrate (Figure S8A–C). Mo-terminated edges readily produce oxygenated Mo. Because the Mo–O bond length (ca. 2.1 Å) [23] is shorter than that (ca. 2.4 Å) of Mo–S, additional *ε*_T_ should exist near the substitution defect sites, facilitating initial unzipping. Proceeding to zz unzipping seems to be associated with abundant S vacancies in CVD-grown MoS_2_ [48], which are expected to be much higher than the S vacancies present in exfoliated MoS_2_ (i.e., ranging from 5 × 10^12^ to 5 × 10^13^ cm^−2^) [49]. The S vacancies accumulate and are transformed into O-substituted defects with high density up to 1 × 10^15^ cm^−2^ upon long-term exposure to ambient conditions [25]. Furthermore, the transmission electron microscopy study [50] showed that, at a high e-beam dose, S vacancies are formed owing to the excision of S atoms. Then, ac line defects up to a few tens of nanometers form as a result of the accumulation of S vacancies by adjacent S diffusion in the MoS_2_ sheet before forming a zz unzipping. Therefore, the formation and accumulation of S vacancies represent a possible model for the formation of directional unzipping near the center. Directional unzipping change from ac to zz is likely to be associated with a larger *ε*_T_ in the basal plane than at the peripheries.

## 4. Conclusions

In summary, in the study described above, we found that initial armchair-directional line defects and subsequent zigzag-directional line unzipping occurred upon treatment with O_2_. Moreover, we showed that these phenomena originate from tension in the chemical-vapor-deposition-grown monolayer MoS_2_ crystals, caused by the thermal expansion coefficient difference with the substrate. The O_2_-treated MoS_2_ crystal exhibited armchair-directional line defects, and the inclusion of water in the incubation atmosphere resulted in further unzipping and folding of MoS_2_. Raman and photoluminescence spectroscopic studies revealed that different prevailing tensions exist in MoS_2_ grown by CVD under the four different conditions. Oxygenated defects, along with tension, further facilitated zigzag line unzipping in the MoS_2_ basal plane upon long-term exposure to an O_2_ atmosphere. The observations provide a potential strategy for directionally selective engineering of the MoS_2_ basal plane as part of efforts to prepare novel building blocks such as MoS_2_ nanoribbons [51]. In addition, the analysis developed for assessing the net contributions of O_2_ and H_2_O utilizing a strain-doping plot should be useful for the understanding of redox and catalytic effects.

## Data Availability

Not applicable.

## Supplementary Materials

The following supporting information can be downloaded at: https://www.mdpi.com/article/10.3390/nano12101706/s1. Figure S1: Schematics of environmental incubations for MoS_2_-containing substrates; Figure S2: AFM phase images of the sample; Figure S3: AFM height images of entrapped water; Figure S4: A° and A^−^ contributions to PL spectra from central regions of MoS_2_; Figure S5: PL spectrum analysis of the peripheral regions of MoS_2_ treated by different environments; Figure S6: PL image and AFM phase image of MoS_2_ before and after O_2_-75RH treatment for three months; Figure S7: The ac-to-zz directional unzipping change, proceeding from edges to center; Figure S8: EDS of selected area from zz unzipped samples.
