# Supplementary material for: Long-Term Exposure of MoS2 to Oxygen and Water Promoted Armchair-to-Zigzag-Directional Line Unzippings
Source: Nanomaterials (Basel). 2022 May 17;12(10):1706. doi: 10.3390/nano12101706 (PMC9145627; doi:10.3390/nano12101706)

Supporting Materials for

Long-Term Exposure of MoS_2 to Oxygen and Water Promoted Armchair-to-Zigzag-Directional Line Unzippings

Youngho Song,[1,†] *Minsuk Park,*[1,†] *Junmo Park,*[1] *Hyun S. Ahn,*[1] *Tae Kyu Kim,*[1] *and Sang-Yong Ju*[1,*]

[1]Department of Chemistry, Yonsei University, Seoul 03722, Republic of Korea

[†] These authors contributed equally to this work.

[*] Correspondence: syju@yonsei.ac.kr

Table of Contents S1

Figure S1. Schematic of environmental incubations for MoS_2-containing substrates S2

Figure S2. AFM phase images of the sample S2

Figure S3. AFM height images of entrapped water S3

Figure S4. A° and A^- contributions of PL spectra from central regions of MoS_2 .... S3

Figure S5. PL spectrum analysis of the peripheral regions of MoS_2 treated by different environement . S4

Figure S6. PL image and AFM phase of MoS_2 before and after O_2-75RH treatment for three months .. S5

Figure S7. ac to zz directional unzipping change by proceeding from edges to center S6–S7

Figure S8. EDS of selected area from zz unzipped samples S8

Figure S1. Schematic of environmental incubations for MoS_2-containing substrates.

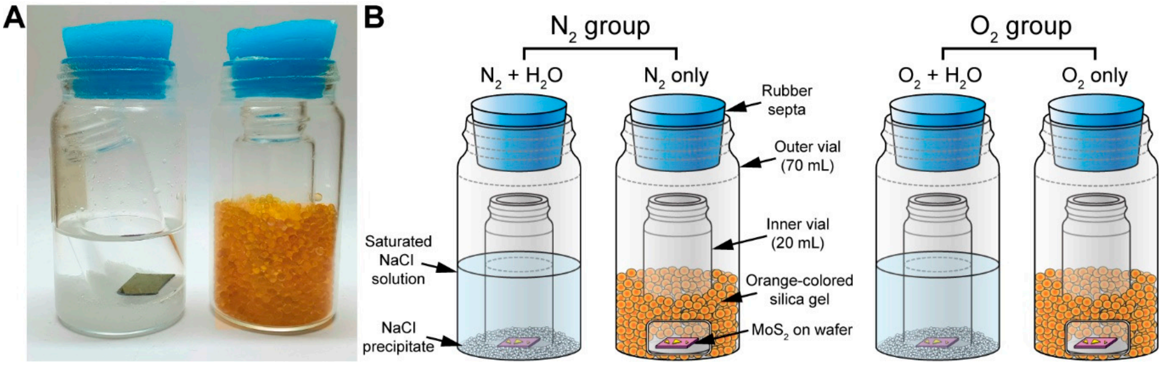

Figure S2. AFM phase images of the sample treated with (A) N_2, (B) N_2-75RH, (C) O_2, and (D) O_2-75RH. Scale bar: 10 μm.

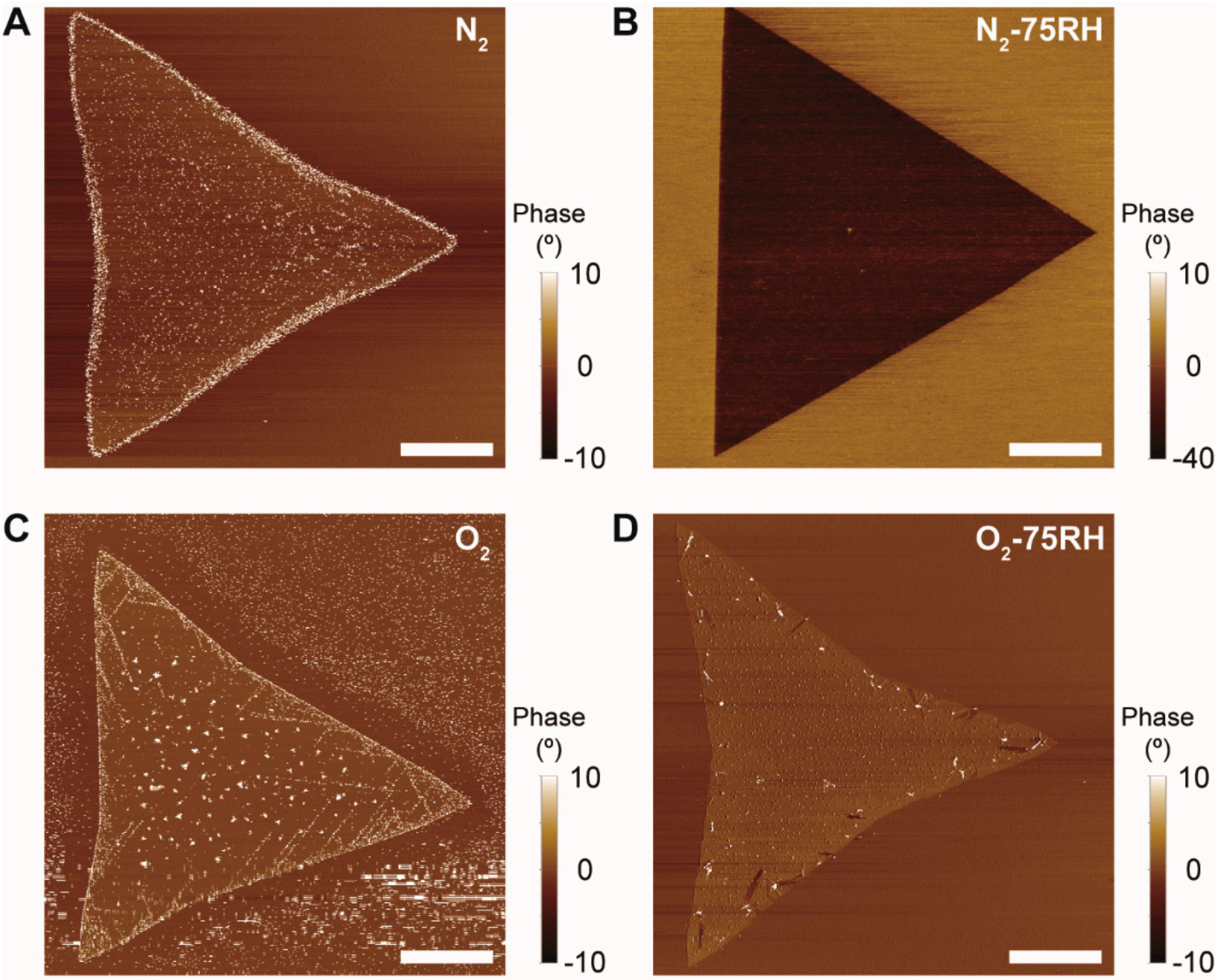

Figure S3. (A) AFM height images of entrapped water between MoS_2 and substrate from N_2-75RH sample, and (B) height profile along the red line in (A).

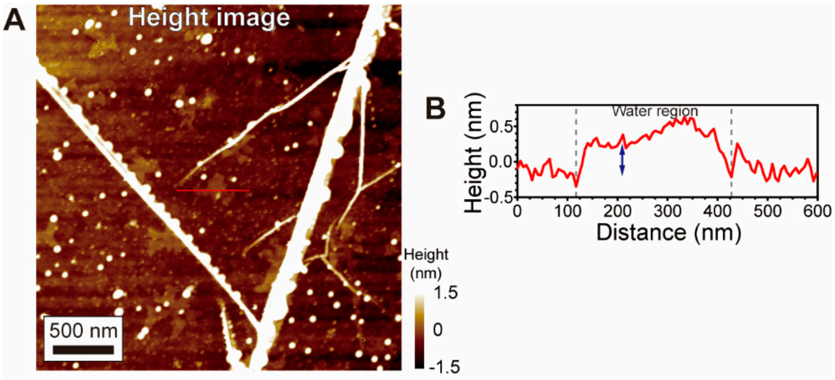

Figure S4. A° and A⁻ contributions of PL spectra from central regions of MoS_2 by each treatment obtained by deconvolution.

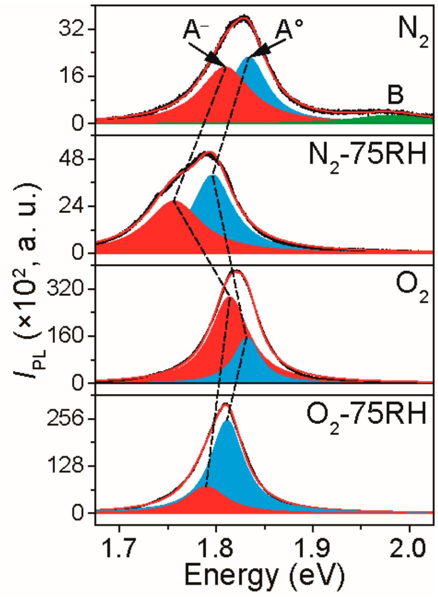

Figure S5. PL spectrum analysis of the peripheral regions of MoS_2 treated by different environement. (A) A° and A^- contributions of MoS_2 by each treatment obtained by deconvolution. (B) Peak position and (C) Peak area changes of A° and A^- derived by each treatment.

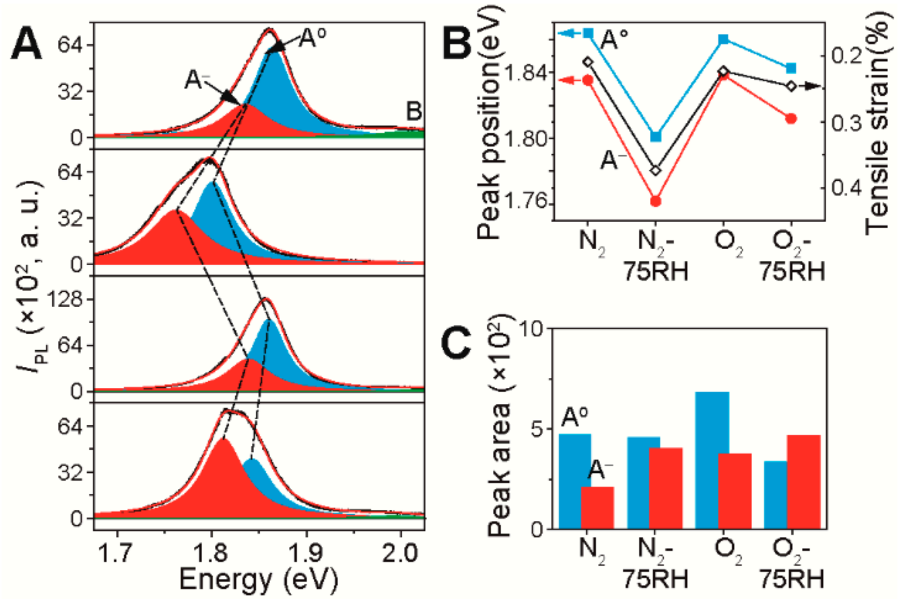

Figure S6. PL image and AFM phase of MoS_2 before and after O_2-75RH treatment for three months. PL images of (A) before and (B) after the treatment. (C) AFM phase image after the treatment.

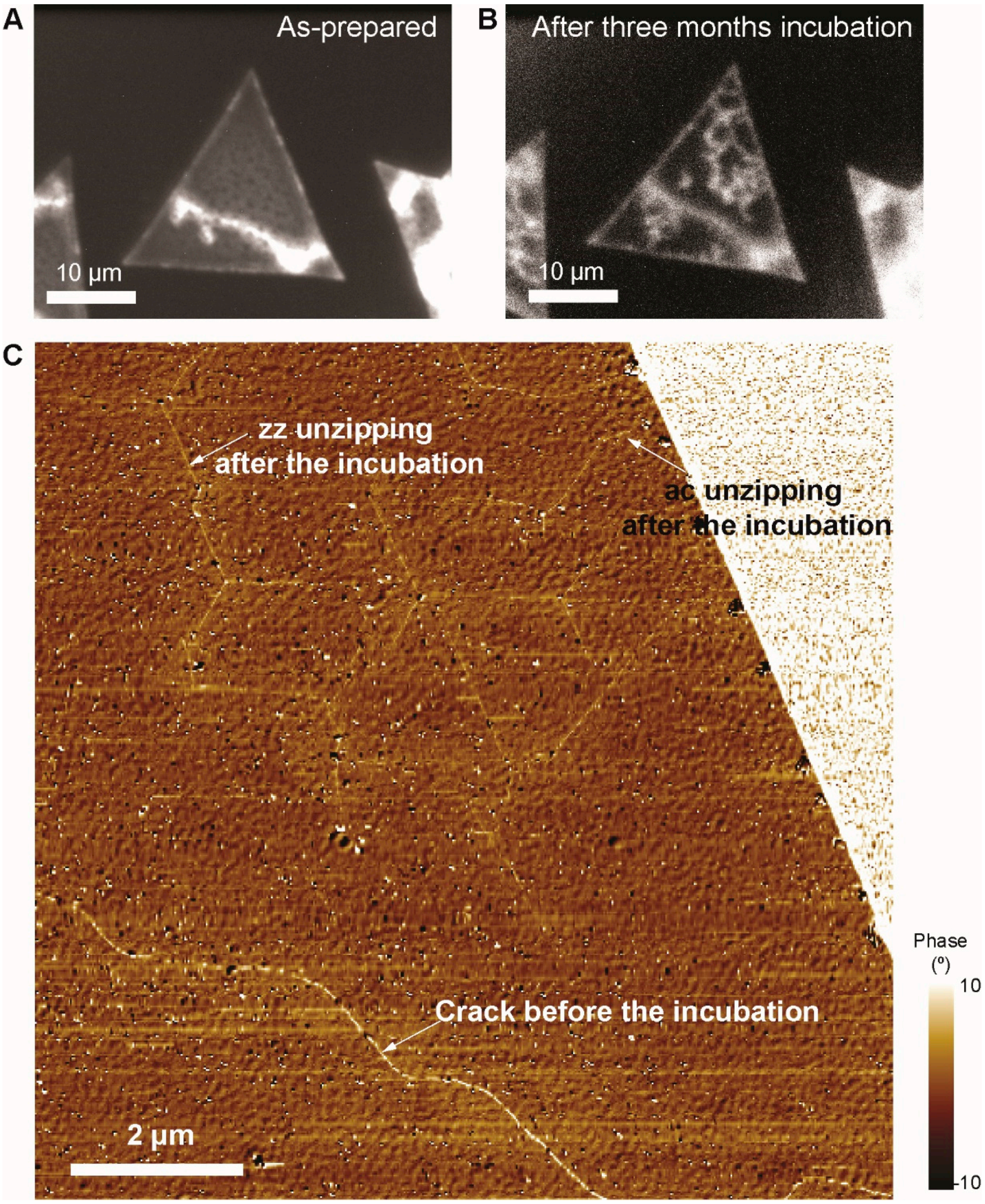

Figure S7. ac to zz directional unzipping change by proceeding from edges to center. AFM phase images of (A–C) O_2-treated and (D–E) O_2-75RH-treated samples. Left image is whole crystal and middle one is zoomed-in image of yellow enclosure in left one. Right is corresponding OM image with indication of number of layers (SL, FL and ML for single, few- and multi-layered MoS_2).

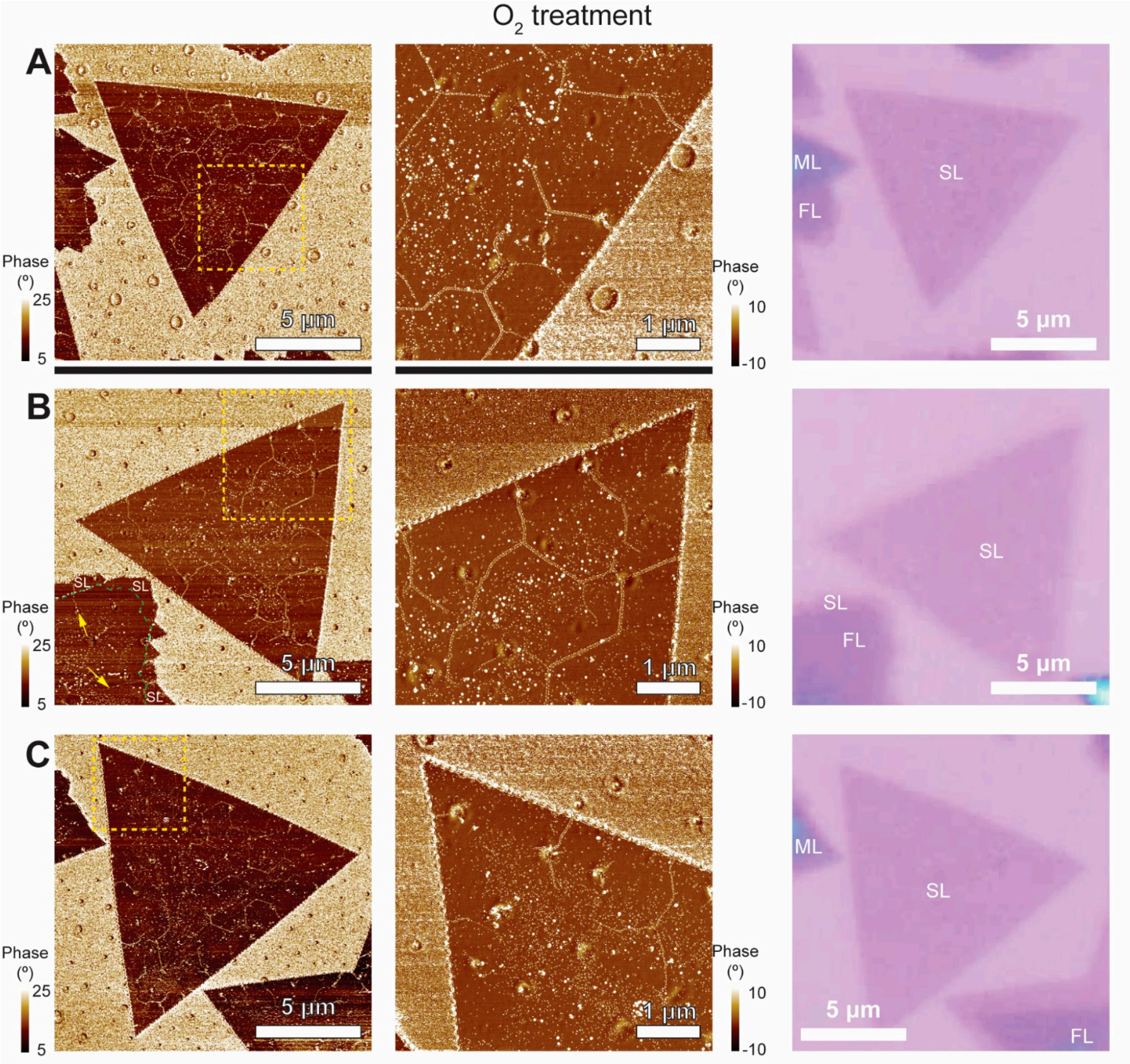

O_2-75RH is continued to the next page

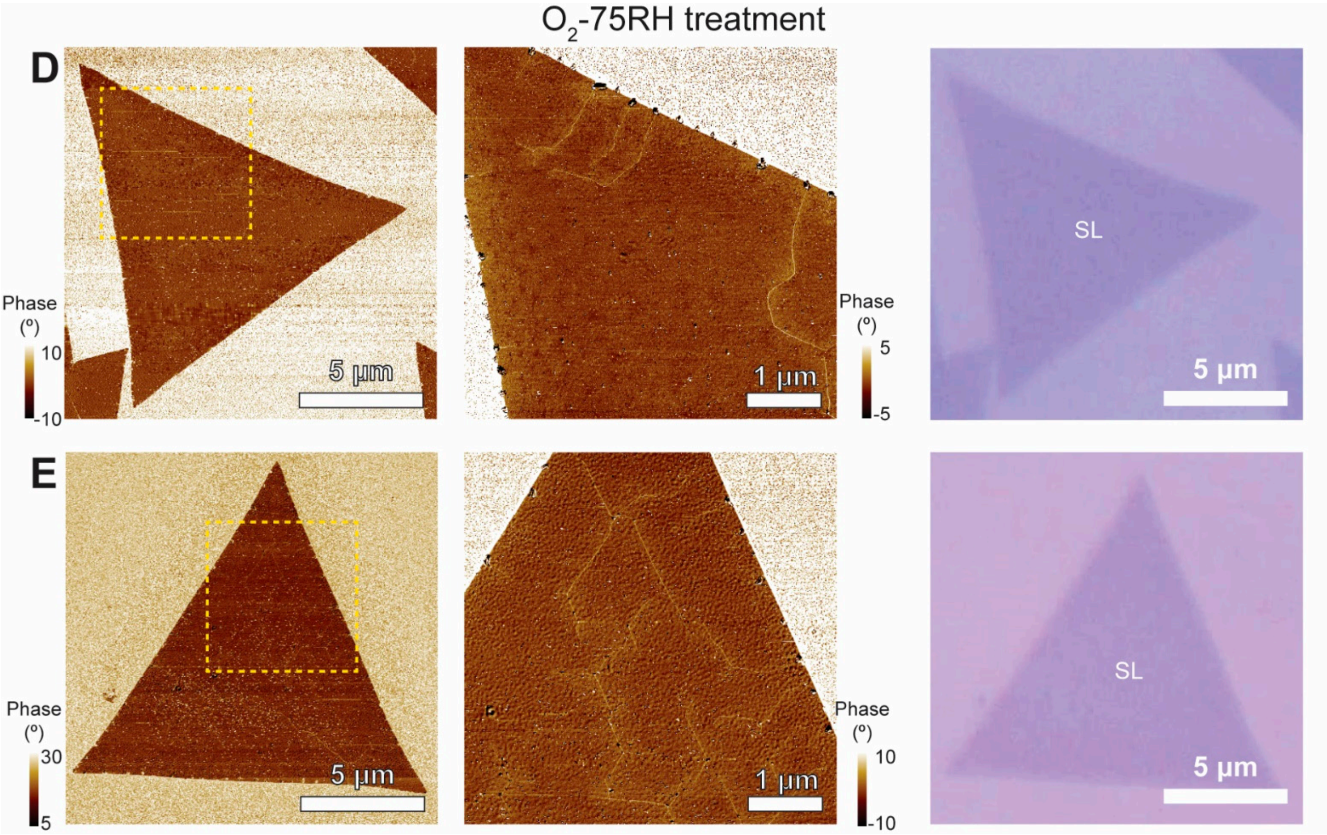

O2-75RH treatment
D
Phase
(º)
10
-10
5 μm
1 μm
Phase
(º)
5
-5
SL
5 μm
E
Phase
(º)
30
5
5 μm
1 μm
Phase
(º)
10
-10
SL
5 μm

Figure S8. EDS of selected area from zz unzipped samples by using scanning electron microscopy (SEM). (A,C) Basal area and (B) zz line defects area. Left one is SEM image, right one is EDS of selected area marked in left image. Inset of right figure lists atomic % present in the samples.

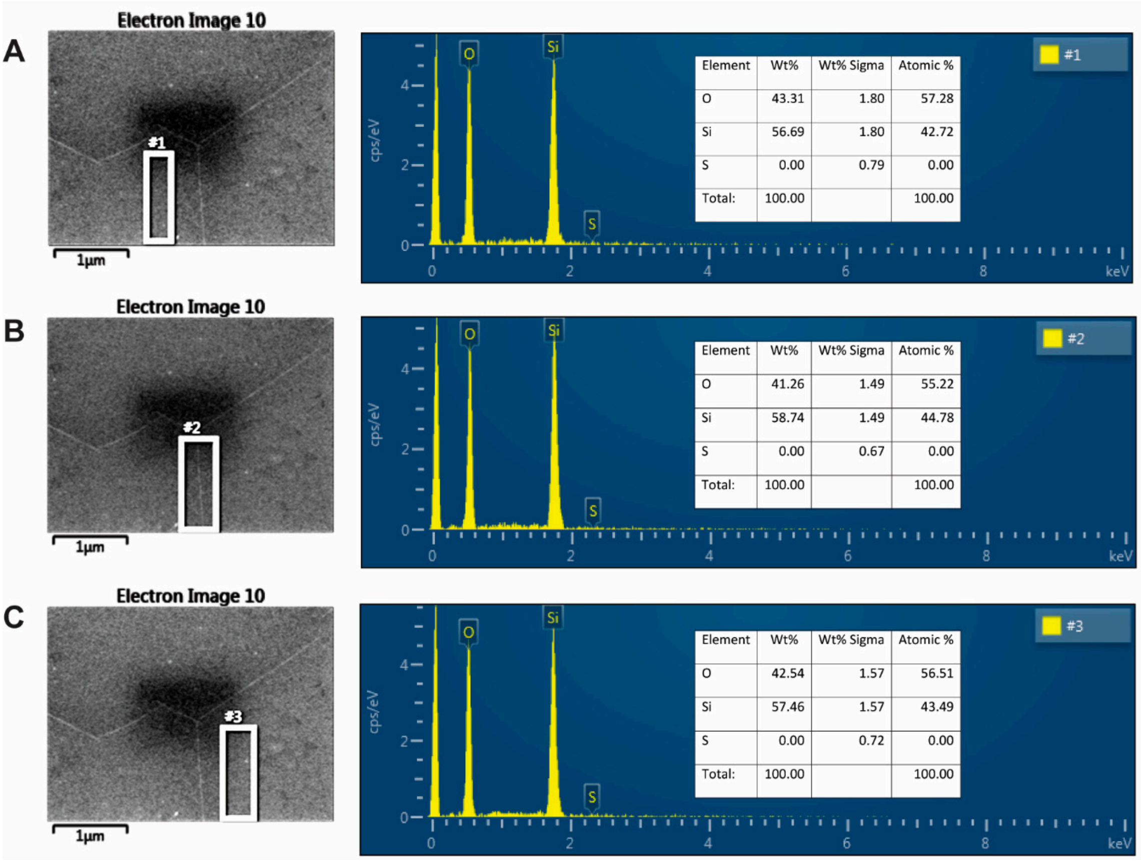

Supplement: Supplementary file 1 [file nanomaterials-12-01706-s001.zip › nanomaterials-1731658-supplementary.pdf]
